# 8.L. Round table: Breaking the barriers: gender equality and women empowerment in public health practice

**DOI:** 10.1093/eurpub/ckac129.516

**Published:** 2022-10-25

**Authors:** Organised by: EUPHAnxt, EUPHA-PHPP, EUPHA SDG5WG, Chair persons: Sofia Ribeiro (EUPHA-PHPP), Jinane Ghattas (EUPHAnxt)

## Abstract

Gender equality is an issue in the public health arena. Though women make 70% of the healthcare workforce, there is an average 28% pay gap. (WHO, 2019). Other barriers have been identified in the literature. (Lancet, 2019) Work-life balance, gender discrimination, sexual harassment or assault in the workplace are pointed out in research studies. Consequences of poor work-life balance include insufficient time with families, difficulties in handling work and all household responsibilities, affecting childbearing decisions. Women also decline leadership opportunities, such as promotions and committee chair positions, because of family obligations. Gender discrimination included feeling inferior and discouragement from promotions or leadership positions on the basis of gender. Another identified barrier was the lack of a safe and unbiased system for seeking help following harassment or assault. Issues related to work-life balance became even more apparent during the COVID-19 pandemic, which placed a disproportionate burden in female public health workers. In the recovery phase, we have an opportunity to rethink public health delivery in order to make it a more equal, less biased, and safe place for women. This should be a concerted effort, involving men and vulnerable populations such as trans women and underrepresented ethnic groups, to ensure that no one is left behind. As one of the leading public health organizations in Europe, the European Public Health Association is committed to join efforts to address this issue in the multiple public health arenas: public health practice, policy and research. This panel discussion is a collaboration between the EUPHA Working Group on gender equality and women's and girl's empowerment, the Policy and Practice section and EUPHAnxt, and is for any conference participant that is committed to reducing the gender gap in public health. The aim of this panel discussion is two-fold. First, we aim at discussing the barriers that female healthcare workers face on an everyday basis, and their impact on their careers. Secondly, we aim at discussing how institutions and individuals can address these barriers and contribute to enhanced gender equality in the public health arena. Following panelists’ interventions (additional speakers have been invited and will be confirmed at a later stage), the audience will be invited to participate in a discussion on gender barriers they have experienced and how those could be addressed.

**Key messages:**

• Public health practice still faces several barriers to gender equality.

• Identifying barriers to gender equality and discussing strategies to overcome them is a step towards achieving gender equality in the workforce.

**Speakers/Panellists:**

**Marie Guichardon**

EUPHA

**Marleen Bekker**

EUPHA-PHPP

